# Management of groin hernias in emergency setting: differences in indications and outcomes between laparoscopic and open approach. A single-center retrospective experience

**DOI:** 10.1007/s00423-024-03238-7

**Published:** 2024-01-26

**Authors:** V. Sbacco, N. Petrucciani, G. Lauteri, A. Cossa, M. Portinari, A. Brescia, G. Garulli

**Affiliations:** 1 Department of Medical and Surgical Sciences and Translational Medicine, Unit of Colorectal Oncologic Surgery, “Sant’Andrea” University Hospital, Sapienza University of Rome, Rome, Italy; 2 Unit of General and Emergency Surgery, Infermi Hospital, Rimini, Italy

**Keywords:** Groin hernia, Strangulated hernias, Minimally invasive surgery, Transabdominal preperitoneal repair, Emergency surgery

## Abstract

**Purpose:**

The use of minimally invasive groin hernia repair techniques in an emergency setting is still debated and its widespread is limited.

The aim of this study is to evaluate the safety and efficacy of the laparoscopic transabdominal preperitoneal (TAPP) technique in the treatment of inguinal and femoral hernias in emergency setting based on our experience, comparing indications and outcomes with the open technique.

**Methods:**

A retrospective analysis was performed including all patients with incarcerated and/or strangulated groin hernia who underwent emergency surgery from November 2019 to September 2022.

Perioperative variables and short- and long-term outcomes were examined.

Statistical analysis was performed using chi-square test for nominal variables and Student’s *t* test for continuous ones. A *p* value < 0.05 was considered statistically significant.

**Results:**

Sixty-six patients were included: 29 patients were treated with TAPP technique (Tapp group) and 37 with open technique plus diagnostic laparoscopy (Open group). Patients in the TAPP group were younger, had less severe clinical scenarios, and had a trend for lower Charlson Comorbidity Index, whereas ASA score and BMI were similar. The small bowel was more frequently herniated in the open group.

Bilateral hernia repair was performed in 20.69% of patients in the Tapp group versus 0% in the Open group (*p* = 0.004). Bowel resection was more frequent in the open group (48.65% vs 0% of the Tapp group, *p* < 0.001) length of surgery was comparable in the two groups. In the Tapp group, the length of hospitalization was significantly shorter (2.59 ± 2.28 days vs. 9.08 ± 14.48 days; *p* = 0.023).

Postoperative complications, according to Clavien-Dindo, were more severe in Open group where there were two deaths. There were no differences in the number of readmission and re-operations at 30 days and in the recurrence rate.

**Conclusions:**

Emergency repair of inguinal and femoral hernias using TAPP is a valuable option, safe and feasible in selected patients. In this series, indications for TAPP were reserved to younger patients with less comorbidities and less severe clinical scenario. Future randomized studies are needed to compare TAPP with open emergency hernia surgery in all settings. Potential advantages of TAPP are the reduction of postoperative complications, earlier recovery, and the possibility of bilateral treatment.

## Introduction

The treatment of hernias of the groin region is one of the most frequently performed surgical procedures in operating theatres around the world. In Italy in 2019 (pre-pandemic period) more than 113,000 inguinal and femoral hernia repair operations were reported [1]. Thanks to the advances in minimally invasive surgery, in the last 20 years the elective surgical treatment for groin hernia has considerably changed and evolved and nowadays the laparoscopic approach, both with TAPP (Transabdominal Pre Peritoneal) and TEP (Totally Extraperitoneal) hernia repair technique, is considered equivalent and even superior to the classic open approach [2, 3]. These two mini-invasive techniques reduce postoperative pain, accelerate the resumption of daily and work activities, and are associated to the same recurrence rate at 5 years compared to the open approach [4, 5].

Hernias can also present in an acute setting; in particular, it is estimated that the risk of strangulated hernia can be as high as 2.9%, thus representing the second leading cause of small bowel obstruction [6, 7]. Obviously, surgery for incarcerated/strangulated hernia has higher postoperative morbidity and mortality than the elective surgical treatment, with higher risk of bowel resection and greater technical difficulties due to several factors including advanced patient age (frequently) and compromised patient’s general condition.

While in elective surgery minimally invasive techniques for hernias repair of the groin region are more and more accepted and used, in the emergency setting their application is still much debated and the available literature on this subject is very limited [8, 9]. Even the current guidelines do not express themselves decisively on this subject [10].

The aim of this retrospective study is to evaluate the indications, the safety, and efficacy of the laparoscopic transabdominal preperitoneal (TAPP) technique in the treatment of inguinal and femoral hernias in emergency setting comparing the minimally invasive treatment with the open technique.

## Material and methods

### Study design, setting, variables, and data sources

All cases of inguinal/femoral hernia undergoing emergency surgery from November 2019 to September 2022 at a Community General Hospital were retrospectively reviewed from a prospective maintained database based on the hospital’s electronic medical records.

All surgeons involved were senior surgeons who had completed their training with the laparoscopic technique. Data protection and privacy were guaranteed and no individual patient could be identified. Information was obtained from the hospital’s digitalized medical registries managed through the Log 80 program (Log80 S.r.l, Healthcare Division) and concerned: gender and age of patients, body mass index (BMI), American Society of Anesthesiology (ASA) score, Charlson Comorbidity Index (CCI), type of surgery, need for bowel resection, length of surgery, length of hospitalization, postoperative complications, reinterventions at 30 days and hospital readmission at 30 days, recurrence, and length of follow-up. The 30-day mortality was also evaluated.

Postoperative complications were assessed according to the Clavien-Dindo (CDC) classification [11] based on outpatient visits records and information obtained through telephone call contact with patients. Outpatient appointments were scheduled in the first week after surgery, after approximately 2 weeks and 1 month.

Follow-up took place with outpatient visits and by telephone contacts.

### Preoperative management

All patients were evaluated in the Emergency Department by the on-call surgeon who analyzed medical history, clinical presentation, blood tests, and clinical examination. In cases of patients presenting with peritonitis or septic shock, emergency surgery was performed directly. In less severe cases, hernia reduction was attempted and/or a CT scan of the abdomen was carried out. Before going to the operating theater, informed consent for hernia surgical repair was collected. The informed consent contained a description of all techniques we used and specified the possibility of bilateral repair if it was necessary and feasible.

### Surgical technique for laparoscopic approach

Surgical treatment was at the surgeon’s discretion each time, with a preference for the TAPP technique except in cases where the patients’ comorbidities did not allow the laparoscopic approach; there was frank peritonitis on clinical examination or emergency CT scan. No indication algorithm was used even because for emergency hernia repair it does not exist yet [12]. All procedures were performed under general anesthesia. A prophylactic dose of antibiotic (30–60 min before the surgery) was administered according to the hospital’s protocol. Locoregional anesthesia using transversus abdominis plane (TAP) block was also performed. Pneumoperitoneum at 12 mmHg was induced using the Hasson technique and, after careful exploration of the abdominal cavity by means of a 30° optic inserted into a 10-mm trocar at the umbilical level, two more 5-mm trocars were placed in the right and left flank approximately at the transverse umbilical line. The preperitoneal space was subsequently created by the incision of the parietal peritoneum of the hernia side from the anterior superior iliac spine to the umbilical artery. The hernia was then cautiously reduced in the abdomen by dissecting the hernia sac from any adhesions respecting the elements of the funiculus. After reducing the hernia in the abdomen, the creation of the prosthesis pocket was completed up to the visualization of the Cooper’s ligament. A self-fixing monofilament polyester mesh (ProGrip™; Medtronic, Minneapolis, MN, USA) was used and secured in all cases to the Cooper ligament with CapSure™ (BD; New Jersey, NJ, USA). The parietal peritoneum was sutured by continuous suture with a self-locking suture.

### Surgical technique for open approach

All procedures were performed under general anesthesia and with prophylactic dose of antibiotic 30–60 min before surgery. The incision was a 5–8-cm inguinotomy depending on the side of the pathology. Afterward, a Lichtenstein Tension-Free Hernia Repair was performed with opening and exploration of the hernia sac, reinforcement of the transversalis fascia, and placement of a polypropylene prosthesis (Angimesh; Angiologica B.M. S.r.l.; Pavia, PV, Italy) secured to the pubic tubercle. If the surgical field was contaminated due to ischemia or perforation of the herniated structures, herniorrhaphy without prosthesis according to the Shouldice’s technique was performed.

Exploratory laparoscopy was additionally performed in cases where there was suspicion of small bowel ischemia at imaging, impossible to evaluate by the inguinotomy (spontaneous reduction of the hernia content after general anesthesia). For the laparoscopy we used one 10-mm umbilical trocar, and two 5-mm trocar on right and left flank. We entered the abdomen with Hasson technique. If the suspicion was confirmed the surgeon carried out a small bowel resection via a minilaparotomy.

If there was any doubt about the viability of the herniated structures, an indocyanine green (ICG) test was performed with injection of 3–5 ml of ICG based on the weight of the patient. If ileal resection and anastomosis was necessary, these were performed using a mechanical stapler. In case of open resection, umbilical minilaparotomy and resection-anastomosis were performed, again using a mechanical stapler.

### Statistical analysis

Data were expressed as mean ± standard deviation or median (interquartile range, 25–75) or as numbers and percentages. Comparisons were made using the chi-square test for nominal data and Student’s *t* test for continuous data. A *p* value < 0.05 was considered statistically significant. All statistical analyses were performed using SPSS software, version 25.

## Results

From November 2019 to September 2022, 66 patients underwent emergency surgery for incarcerated or strangulated inguinal and/or femoral hernias. Seventy-three percent of the patients were operated on the same day of admission at the Emergency Department.

The cases were divided as follows: 29 patients operated with laparoscopic TAPP technique (Tapp group) and 37 with Lichtenstein technique combined with diagnostic laparoscopy to assess the viability of the bowel tract involved in the herniated defect (Open group); the latter group also includes 12 patients treated only with Lichtenstein tension-free hernia repair.

The mean age was 69 ± 14.43 years in the Tapp group and 77.30 ± 14.46 years in the Open group (*p* = 0.024), with a higher proportion of patients aged > 75 years in the Open group (12 patients versus 25 patients, respectively; *p* = 0.035). There was no significant difference between the two groups either in the male:female ratio or in ASA score and BMI. Patients’ comorbidities were assessed by Charlson Comorbidity Index (CCI), with a score of 3.31 ± 2.25 for the Tapp group and 4.38 ± 2.18 for the Open group (*p* = 0.055). No significant difference was found between the two groups regarding the type of hernia presented. The hernial content was represented by an ileal loop in 27 cases in the Open group, while this occurred in only 7 of the laparoscopically treated cases (*p* = 0.0001). Similar proportion of recurrent hernias were observed in the two groups. All cases of recurrence were previous treated with open technique with mesh placement.

The demographic characteristics of the patients and the preoperative variables are summarized in Table 1.

**Table 1:** Patient’s demographic characteristics and preoperative variables

	Tapp group (*n* = 29)	Open group (*n* = 37)	*p*-value
Age (mean ± DS) and range	69 ± 14.43 (35–92)	77.30 ± 14.46 (34–101)	0.024
≤ 65 (*n*, %)	11 (37.9%)	7 (18.92%)	0.088
66–75 (*n*, %)	6 (20.69%)	5 (13.51%)	0.441
≥ 75 (*n*, %)	12 (41.38%)	25 (67.57%)	0.035
Men/woman	20/9	21/16	0.314
ASA 1 (*n*, %)	2 (6.90%)	2 (5.40%)	0.803
ASA 2 (*n*, %)	13 (44.83%)	9 (24.32%)	0.112
ASA 3 (*n*, %)	14 (48.28%)	23 (62.16%)	0.263
ASA 4 (*n*, %)	0	3 (8.10%)	0.129
BMI (mean ± DS) and range	31.87 ± 21.19 17.3–34.6	36.45 ± 25.43 16.38–39.09	0.446
< 18.5	1 (3.45%)	1 (2.70%)	0.862
18.5–24.9	13 (44.83%)	17 (45.95%)	0.928
25–30	10 (34.48%)	15 (40.54%)	0.617
30.1–34.9	5 (17.24%)	2 (5.41%)	0.124
35–40	0	2 (5.41%)	0.207
> 40	0	0	
Charlson comorbidity index (mean ± DS)	3.31 ± 2.25	4.38 ± 2.18	0.055
Type of hernia (*n*, %)			
Inguinal	17 (4.93%)	15 (40.54%)	0.148
Direct	5 (1.45%)	5 (13.51%)	0.677
Indirect	12 (3.48%)	11 (29.73%)	0.328
Internal oblique	0	1 (2.70%)	0.376
Femoral	6 (1.74%)	14 (37.84%)	0.135
Inguinofemoral	2 (0.58%)	0	0.107
Inguinoscrotal	5 (1.45%)	5 (13.51%)	0.677
Hernia content (*n*, %)			
Omentum	18 (62.07%)	5 (13.51%)	< 0.001
Small bowel	7 (24.14%)	27 (72.97%)	0.0001
Sigma	2 (6.90%)	4 (10.81)	0.586
Right colon	1 (3.45%)	0	0.259
Appendix	0	1 (2.70%)	0.376
Meckel’s diverticulum	0	1 (2.70%)	0.376
Recurrent hernia	8 (2.32%)	7 (18.92%)	0.408

Locoregional anesthesia using transversus abdominis plane (TAP) block was also performed in 21 of 29 patients (72.4%) of the Tapp group. Herniorrhaphy without prosthesis according to the Shouldice’s technique was never performed in the TAPP group while was performed in 37.84% of the patients in the Open group (*p* = 0.0002). The laparoscopic approach allowed a bilateral repair in 20.69% of the cases versus 0% in the Open group (*p* = 0.004). The bowel resection rate was 0% and 48.65% in the Tapp and Open groups, respectively (*p* ≤ 0.0001). Of the 18 total resection of the Open group, 15 (40.54%) were made by laparotomy and 3 (8.11%) by laparoscopy. In each case the resection concerned the small bowel. There was no case of conversion in the Tapp group. The length of surgery was 109.55 ± 50.53 min in the Tapp group and 98.69 ± 42.94 in the Open group (*p* = 0.343). No significant difference was detected in the operating time even comparing the duration between laparoscopic repair and the 12 patients treated with classical anterior technique without diagnostic laparoscopy (109.55 ± 83.88, *p* = 0.134). Details about length of surgery of unilateral and bilateral TAPP are reported in Table 2. The length of hospitalization was significantly shorter in the Tapp group than in the Open group (2.59 ± 2.28 days vs. 9.08 ± 14.48 days; *p* = 0.023).

**Table 2:** Intraoperative parameters and postoperative hospitalization

	Tapp group (*n* = 29)		Open group (*n* = 37)	*p*-value
Length of surgery (min; mean ± DS)	109.55 ± 50.53		98.59 ± 42.94	0.343
			Open repair without diagnostic laparoscopy (*n* = 12) 83.88 ± 44.43	0.134
Length of surgery (min; mean ± DS)	Unilateral TAPP 94.17 ± 39.74	Bilateral TAPP 173.25 ± 58.5		0.002
Type of repair (*n*, %)				
Herniorrhaphy without prosthesis	0		14 (37.84%)	0.0002
Prosthetics	29 (100%)		23 (62.16%)	0.0002
Monolateral	23 (79.31%)		37 (100%)	0.004
Bilateral	6 (20.69%)		0	0.004
Small bowel resection in total (*n*, %)	0		18 (48.65%)	< 0.0001
Open small bowel resections	0		15 (40.54%)	
Laparoscopic small bowel resections	0		3 (8.11%)	
Length of hospitalization (days)				
Mean ± DS	2.59 ± 2.28		9.08 ± 14.84	0.023
Median and interquartile range	2 (1–3)		2 (1–8.5)	
Bowel perforation (*n*, %)	0		3 (8.11%)	0.119

Data on intraoperative variables and hospitalization are reported in Table 2.

In our series, the total complications of the laparoscopic approach were 55.17% (16 of 29) and as a type completely overlapping with those reported by previous studies [9, 13, 14]. There was a prevalence of grade I complications according to Clavien-Dindo, and the only three cases of higher grade (Clavien-Dindo IIIa and IIIb) were an intraabdominal collection drained by interventional radiology procedure, an incisional hernia operated on 1 year later, and an acute appendicitis operated on a few days after hernia repair.

In the Open group, complications were 51.35% (19 out of 37) but we had some grade IVa, IVb, and V complications including heart failure and pneumonia, two episodes of wound dehiscence, and two deaths from causes related to the pathology and the surgery (hemorrhagic shock and intestinal evisceration with perforation).

The only significant difference observed concern grade II complications: 0 cases in the Tapp group vs. 5 cases (13.51%) in the Open group with *p* = 0.041.

Mortality rate was 0% in Tapp group and 5.41% (2 cases) in the Open group (*p* = 0.219), both related to the complications resulting from the operation.

There were no significant differences in the number of hospital readmissions (10.35% for the laparoscopic group and 13.51% for the open group) and no differences in number of reinterventions at 30 days. There were no significant differences in the recurrence rate. In the Tapp group, where all repairs were mesh-based we observed 2 recurrences, in the Open group we detected a total of 3 recurrences all in patients that had a mesh-based repair (6.90% vs. 8.11%; *p* = 0.855).

Follow-up lasted an average of 17 months. A total of two patients died during the follow-up period and none of them had shown recurrence of hernia before death.

Surgical outcomes are shown in Table 3.

**Table 3:** Surgical outcomes

	Tapp group (*n* = 29)	Open group (*n* = 37)	*p*-value
30-day readmission (*n*, %)	3 (10.35%)	5 (13.51%)	0.698
30-day reintervention (*n*, %)	1 (3.45%)	4 (10.81%)	0.291
Recurrence (*n*, %)	2 (6.90%)	3 (8.11%)	0.855
Time of recurrence (months)			
Mean ± DS	14.5 ± 16.26	21.3 ± 7.09	0.026
Follow-up (months; mean ± DS)	13.33 ± 11.11	16.54 ± 11.31	0.253
Complications according to Clavien-Dindo (*n*, %)	16 (55.17%)	19 (51.35%)	0.759
No complications	13 (44.83%)	18 (48.65%)	0.177
I	13 (44.83%)	6 (16.22%)	0.087
II	0	5 (13.51%)	0.041
IIIa	1 (3.45%)	0	0.259
IIIb	2 (6.90%)	2 (5.41)	0.803
IVa	0	3 (8.11%)	0.119
IVb	0	1 (2.70%)	0.376
V	0	2 (5.41%)	0.207
Mortality (*n*, %)	0	2 (5.41%)	0.219

## Discussion

Over the years, the laparoscopic approach has become the gold standard for the emergency treatment of many conditions, including acute appendicitis and acute cholecystitis; however, the laparoscopic treatment of incarcerated or strangulated groin hernias, one of the most frequent causes of emergency department access worldwide, is still debated [15]. Latest WSES guidelines are still cautious about this topic and suggested laparoscopic approach only in the absence of strangulation [16] and the HerniaSurge group underline that no randomized study focus on laparoscopic technique in emergency [12].

The advantages of minimally invasive techniques for the treatment of hernias are well known and are mainly relate to lower rate of wound infection, better cosmetic results, reduced hospital stay, possibility of bilateral treatment in a single time with the same access, less postoperative pain, and faster return to work [2, 3]. Many meta-analyses, randomized trials, and several guidelines indicate that laparoscopic surgery in the elective setting is comparable to the open approach and in many aspects even more advantageous [5, 17, 18].

In the field of emergency surgery, although the first laparoscopic operation for strangulated inguinal hernia was performed in 1993 [19], still today there is no uniform consensus on the use of minimally invasive techniques [15, 20].

The aim of our study is not to compare laparoscopic and open techniques in terms of outcomes, as our criteria to perform emergency open or laparoscopic surgery for groin hernia were different. Our aim is to evaluate the differences in preoperative indications for both procedures, and to report the results of both techniques. The present study is not a randomized study and it is evident that TAPP was performed to treat patients with less severe disease (younger, with lower CCI, with no peritonitis). Furthermore, we aim to provide new data for the evaluation of the role of laparoscopy in the treatment of hernias of the groin region in the emergency setting. We therefore compared the transabdominal preperitoneal technique with the classic open technique to understand the feasibility and safety of the former. Feasibility was assessed by taking as endpoints the length of surgery, rate of bowel resections, rate of conversions, and length of hospitalization; to assess safety, on the other hand, we considered complications, possible readmission, and reinterventions at 30 days, number of recurrences, time to recurrence, and mortality.

For the 29 patients of the laparoscopic group, the length of surgery was slightly longer than for the 37 patients of the open group (109.55 ± 50.53 vs. 98.59 ± 42.94), with a nonsignificant difference. It should be noted that in our series, patients treated with the open technique often underwent diagnostic laparoscopy at the end of surgery to assess the viability of the loop, as the guidelines recommend [10, 16]; this certainly increased the length of surgery. However, if we compare the Tapp group with the subgroup of open patients for whom diagnostic laparoscopy was not performed, we still obtain a nonsignificant difference, demonstrating that in the hands of experienced surgeons the TAPP technique is not excessively time-consuming [8].

In our experience, the number of bowel resections was much higher in the open group with 18 resections versus 0 in the Tapp group. We need to highlight that the open group had more often a severe presentation and in 72.97% of the cases there was an intestinal loop involved in the hernia defect, while in the Tapp group the hernia content was almost always represented by omentum. There were no intraoperative complications typically related to laparoscopy, such as bowel or vascular injuries often associated with the introduction of trocars. Our conversion rate was zero, proving to be lower than that currently reported in the literature [9, 21].

Laparoscopic repair allows the surgeons to be able to judge the viability of the bowel loops possibly involved in the herniated defect: during the procedure we visually monitor the ischemic loops evaluating both color and peristaltic activity of the ileal loops; furthermore, the whole phase of hernia reduction and prosthesis placement can be helpful for the intestinal loop to recover, avoiding unnecessary bowel resections [19, 20]. If the resection is necessary and is performed laparoscopically, in our experience the bowel is manipulated only after the prosthesis is placed and the parietal peritoneum is closed. If, on the other hand, it is performed by minilaparotomy, the periumbilical site is usually chosen as access. This separation of procedures and surgical wounds certainly reduces the infection rate of the mesh and the wounds themselves [9, 20].

Historically mesh placement was avoided when the risk of mesh infection was increased. In the laparoscopic approach where the use of prosthetic mesh is mandatory, we might think that this increased the risk of infection especially in classes III and IV of the CDC surgical wound classification. The latest evidence, however, shows that the use of mesh is safe even in cases of strangulated hernias requiring bowel resections with surgical site contamination [10, 22].

The improved exploration of the abdominal cavity allowed by laparoscopy guarantees the possibility of detecting associated defects, such as contralateral hernias or co-presence of inguinal and femoral hernias [6, 21]. In fact, there were as many as 6 bilateral repairs in the Tapp group versus all unilateral repairs in the open group (*p* = 0.004).

Examining the length of hospitalization, it can be seen, as emerged from other previous studies [9, 23, 24], that this is significantly lower in the Tapp group than in the Open group (2.59 ± 2.28 days vs. 9.08 ± 14.84 days, *p* = 0.023) and our hospitalization length of stay data of 2.59 days is perfectly in line and even lower than that of other similar studies on the same topic [9].

Reduced hospitalization, less postoperative pain, and fewer complications are often associated with faster resumption of work activities, which is reflected in an advantage in the socioeconomic field [18, 25, 26]. The length of stay of the open group could be influenced by the higher bowel resection rate, so randomized trial and bigger studies are necessary to confirm this result.

In our series complications of the open approach were slightly lower than the ones of the laparoscopic approach (51.35% vs 55.17%, *p* = 0.759), but a more critical interpretation of the data leads us to state that the laparoscopic group had less severe complications.

In fact in the open approach we counted some grade IVa, IVb, and V complications, whereas in the Tapp group we had almost completely grade I complications.

The 30-day readmission rate was comparable and was 10.35% for the laparoscopic group and 13.51% for the open group. In the first group, one patient returned to the hospital due to COVID, one had a stroke 1 month after surgery, and one patient was readmitted due to fever not associated with surgical wound infection or abdominal collections. In the second group, the 5 readmissions were due to subarachnoid hemorrhage, dehydration, bowel occlusion, wound infection, and evisceration with bowel perforation for which reintervention was required. We can see that in the open group all patients who returned to the PS had undergone bowel resections, and 2 out of 5 returned for issues related to surgery.

There was also no significant difference in the two groups regarding the 30-day reintervention rate (3.45% in the Tapp group vs. 10.81% in the open group, *p* = 0.291). In the minimally invasive group, the only reintervention was performed for acute appendicitis; in the open group, we recorded one reintervention performed for anastomosis bleeding, two for anastomotic dehiscence of which one was associated with peritonitis and acute appendicitis, and one for evisceration followed by bowel perforation. One of the patients who underwent reintervention subsequently died.

The recurrence rate is only slightly higher for the classic approach (*p* = 0.855); however, when it occurs, it appears earlier in laparoscopically treated patients (14.5 ± 16.26 months vs. 21.3 ± 7.09 months, *p* = 0.026). Has we described above, apparently no one of the patients with direct repair had a recurrence but those patients are the ones with the worse clinical presentation, so most of them died during the operation or soon after because of the age.

Despite the advantages of laparoscopy, the use of the TAPP technique for the treatment of groin hernias in the emergency setting is not systematically recommended by guidelines and we are still far from its routinely use; the reason for this is because it is a complex technique and requires rather long learning curve [27–29].

Our analysis shows that TAPP for incarcerated/strangulated groin hernias has results comparable with the classical technique in terms of operating time, 30-day hospital readmissions, 30-day reinterventions, and recurrence rate. In contrast it has advantages over open surgery, above all for patient without bowel ischemia at presentation, such as reduced length of hospitalization, possibility of bilateral treatment, and less severe complications associated with surgery. We underline that in our experience the laparoscopic technique was reserved for “simpler” patients with less comorbidities and less severe clinical scenarios.

## Limits

Our study certainly has limitations mainly related to the fact that it is a retrospective analysis, the low sample size, and the short duration of follow-up.

The indications for one procedure or the other were different and this was not a randomized trial. A worse clinical presentation prompted surgeons to choose the open approach, which explains worse postoperative outcomes in that group. All the results should be carefully evaluated considering the significant differences in the preoperative patients’ characteristics and disease presentation.

## Conclusions

The treatment of groin hernias in the emergency setting is a challenge that involves every general surgeon throughout his career. The evolution of minimally invasive techniques is leading to their initial use in this setting as well.

The TAPP technique has proven to be feasible and safe in this field when performed by experienced surgeons and in selected patients.

Comparative studies between laparoscopic and open surgery with similar selection criteria and larger numbers of included patients and especially prospective randomized trials are needed to allow more widespread use of this approach.

## Data Availability

The data that support the findings of this study are available on request from the corresponding author.
